# Flexible Piezoresistive Film Pressure Sensor Based on Double-Sided Microstructure Sensing Layer

**DOI:** 10.3390/s24248114

**Published:** 2024-12-19

**Authors:** Rong Sun, Peng Xiao, Lei Sun, Dongliang Guo, Ye Wang

**Affiliations:** 1 State Grid Jiangsu Electric Power Co., Ltd., Research Institute, Nanjing 211103, China; 2 Joint International Research Laboratory of Information Display and Visualization, School of Electronic Science and Engineering, Southeast University, Nanjing 210096, China

**Keywords:** film flexible pressure sensor, double-sided microstructure, pressure sensing, sitting posture detection

## Abstract

Flexible thin-film pressure sensors have garnered significant attention due to their applications in industrial inspection and human–computer interactions. However, due to their ultra-thin structure, these sensors often exhibit lower performance, including a narrow pressure response range and low sensitivity, which constrains their further application. The most commonly used microstructure fabrication methods are challenging to apply to ultra-thin functional layers and may compromise the structural stability of the sensors. In this study, we present a novel design of a film pressure sensor with a double-sided microstructure sensing layer by adopting the template method. By incorporating the double-sided microstructures, the proposed thin-film pressure sensor can simultaneously achieve a high sensitivity value of 5.5 kPa^−1^ and a wide range of 140 kPa, while maintaining a short response time of 120 ms and a low detection limit. This flexible film pressure sensor demonstrates considerable potential for distributed pressure sensing and industrial pressure monitoring applications.

## 1. Introduction

Flexible thin-film pressure sensors are attracting increasing interest due to their considerable potential for applications in healthcare [1,2,3], human–computer interaction [4,5], industrial inspection [6,7], and intelligent robotics [8,9,10,11]. This is due to their superb properties, including thinness, lightness, low cost, and suitability for use with a variety of contact surfaces [11,12,13,14,15,16,17]. Current studies that explore flexible thin-film pressure sensors focus on the sensitivity, sensing range, and response time of the sensors [18,19,20,21]. Compared to sensors with different mechanisms, such as capacitive, piezoelectric, and magnetic mechanisms, piezoresistive sensors exhibit considerable advantages due to their straightforward structure, low interference, sensitivity, and reliability [22,23,24]. These advantages enable the sensors to play a significant role in various scenarios, including intelligent manufacturing, IoTs, smart medical applications, etc.

Conventional resistive pressure sensors are typically constructed with two distinct structures. The first is a double-layer configuration consisting of a functional layer and an interdigital electrode [25,26]. This structure senses pressure through the contact resistance change between the functional layer and the interdigital electrode. The second structure is a sandwich configuration comprising an electrode, a functional layer, and another electrode [27,28]. This structure senses pressure through the contact between the electrode and the functional layer, as well as the resistance of the functional layer during compression. It is essential that the functional layer exhibits a high degree of deformation capability in order to guarantee optimal device performance. However, the size of the thin-film sensor makes it challenging for the ultra-thin intermediate layer to undergo significant deformation, which in turn affects the performance of the device. Consequently, thin-film pressure sensors do not perform well in terms of sensitivity and sensing range. To address these issues, microstructures are frequently employed to enhance the deformation capability of the functional layer, thereby optimizing the pressure response of the sensor. To improve the sensitivity of the sensor, microstructures are formed on the surface of the film using the template array method [29,30], the bionic template method [31,32], and the expanded microsphere solution method [33,34]. These methods can produce compressive deformation under pressure based on the stress concentration effect, thus improving the overall deformation capability of the functional layer film. Furthermore, in order to extend the range of sensors, internal porous structures or surface multi-scale microstructure arrays are frequently employed [35,36]. However, these techniques are difficult to apply in thin-film structure sensors because of their size, and they may lead to structural instability over time, which can shorten the sensor’s operational lifespan and reduce the overall reliability of the devices. Consequently, the construction of microstructures on thin-film-type pressure sensors to enhance the sensor sensitivity and to meet the requirements of a wide range and low response time remains a great challenge. From the perspective of optimizing the compression performance of the functional layer, the integration of surface microstructures with the internal deformation sensing mechanism of the functional layer represents an effective solution.

Herein, we present a novel microstructure transfer method using sandpaper to fabricate double-sided microstructure conductive composite films. Subsequently, a sandwich-structured thin-film pressure sensor was prepared by sandwiching the thin-film package with upper and lower electrodes. The composite film’s excellent mechanical properties were reflected in the pressure sensor’s high sensitivity (5.5 kPa^−1^), wide sensing range (0–140 kPa), short response and recovery time (120 ms/93 ms), and good durability over more than 2000 cyclic loading/unloading tests. Furthermore, based on the pressure sensor, a system for detecting sitting postures was developed to analyze the support of the chair to different sitting postures, with the objective of distinguishing between healthy and unhealthy sitting postures. These results indicate that the sensor has the potential for a wide range of applications.

## 2. Experimental Section

### 2.1. Materials

The conductive carbon black (EC-600JD) was purchased from Nanjing XFNANO Materials Tech Co. Ltd. (Nanjing, China). PDMS (DC 184), which consists of a PDMS prepolymer and curing agent, was purchased from Dow Corning, Inc. (Midland, MI, USA). N-Hexane solution (≥99%) was purchased from Shanghai Macklin Biochemical Technology Co. Ltd. (Shanghai, China). Conductive silver paste (JC-2100) was purchased from Jiecheng Technology Co. Ltd. (Dongguan, China). Polyethylene terephthalate (PET) film (0.2 mm thick) was purchased from Aiqiu Electronic Co. Ltd. (Taizhou, China). The above chemicals and materials were purchased and used directly.

### 2.2. Preparation of Double-Sided Structure Conductive Layer

**Figure 1:**
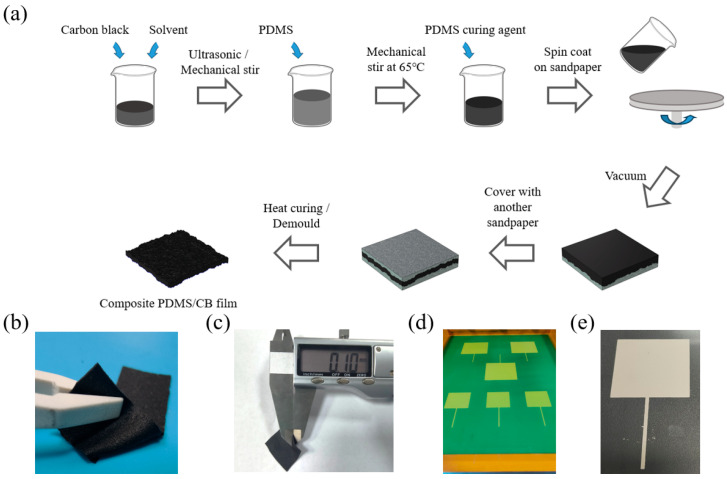
The double-sided composite PDMS/CB film: (**a**) the fabrication process of the double-sided composite PDMS/CB film, (**b**) the optical image of the composite film, (**c**) the thickness of the composite film, and (**d**) the screen-printed electrode stencil and (**e**) electrode.

Figure 1a shows the whole fabrication process of the double-sided microstructure composite film. First, 0.5 g of flaky conductive carbon black (CB) was mixed with 10 mL of n-hexane solvent and sonicated for 30 min, before being magnetically stirred for 30 min until the conductive carbon black was initially dispersed in the solution. Subsequently, 10 mL of polydimethylsiloxane (PDMS) was added to the mixture and mechanically stirred at 65 °C for 30 min to evaporate the solvent. Afterward, 1 mL of the PDMS curing agent was then added to the solution and stirred for 30 min so that the components of the solution were completely dispersed. The mixed solution was then poured into a sandpaper-covered spin coater and spin-coated for 20 s at 800 rpm. After spin coating, the sandpaper and the mixed solution were placed in a vacuum drying oven to evacuate the air, and then another piece of sandpaper was covered with the mixed solution with the rough side downward, after which the mixed solution was completely dried and the sandpaper on both sides was removed to obtain an ultra-thin PDMS/CB composite conductive film with a two-sided microstructure. Figure 1b shows an optical image of the composite film, with the sandpaper-like, rough, and uneven surface structure evenly distributed on both sides of the film.

### 2.3. Preparation of Flexible Pressure Sensor

The electrodes for the sensor were prepared using a screen-printing process. Through a pre-designed screen printing stencil shown in Figure 1d, a square-shaped electrode was printed on a polyethylene terephthalate (PET) substrate film using conductive silver paste, and then the two printed electrodes and the microstructure conductive layer prepared above were combined and packaged in a sandwich structure to obtain the flexible thin-film pressure sensor in this article. As shown in Figure 1c, the thickness of the composite PDMS/CB film was 0.1 mm and the overall thickness of the pressure sensor was 0.3 mm.

### 2.4. Finite Element Analysis (FEA)

COMSOL Multiphysics 6.0 was used for the static simulation of the composite film to investigate the contact stress and deformation with different types of surface structures. Moreover, the 2D module and structural mechanics interfaces were used for static force and post-processing.

### 2.5. Characterization and Measurements

The morphologies of the composite PDMS/CB film were examined using a Tescan mira4 field-emission scanning electron microscope. The electro-mechanical properties of the pressure sensor were measured using a force gauge TST-01H from Pubtester, source meter Keithley 2400, and an oscillometer DSO7034B.

## 3. Results and Discussion

### 3.1. Finite Element Analysis of Mechanical Properties of Composite Film

**Figure 2:**
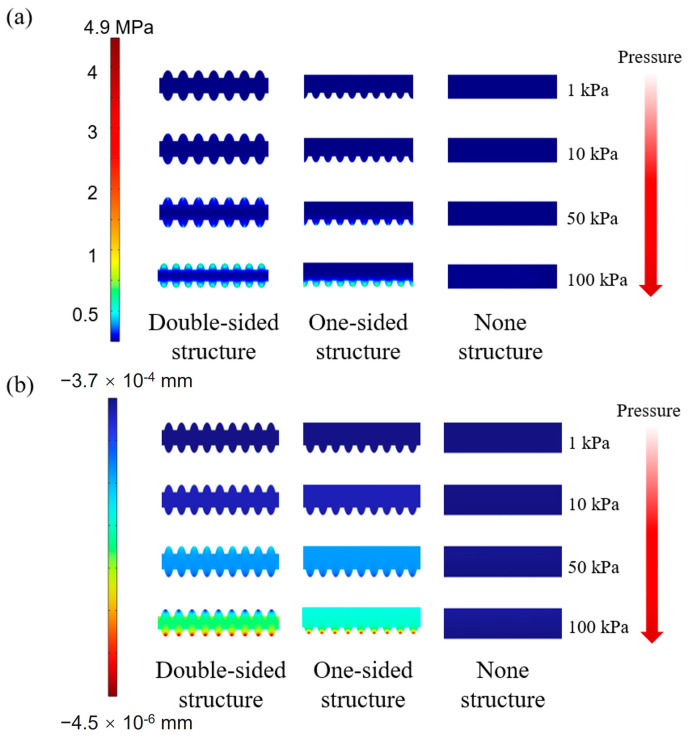
Finite element analysis of double-sided, one-sided, and non-structure films under 1 kPa, 10 kPa, 50 kPa, and 100 kPa: (**a**) stress distribution and (**b**) y-direction displacement.

COMSOL Multiphysics 6.0 (COSMOL) was used for the mechanical simulation of the composite film to investigate the effect of different surface structures on the mechanical properties. All the three films had the same thickness of 0.1 mm. The left film had rough microstructures on both sides, the middle film had microstructures on only one side and the other side was planar, and the right film had no microstructures and was planar on both sides. The density of the composite film was set to 0.97 g/cm^3^, Young’s modulus was 750 kPa, and Poisson’s modulus was 0.49. In this model, the bottom electrode layer was fixed as a constraint, and pressure was applied to the surface of the top electrode, and Young’s modulus of the two electrodes was set at a large level to ignore the electrode deformation. The mechanical simulation result is shown in Figure 2. It can be seen from Figure 2a that at the surface of the film in contact with the electrode, the stresses were clearly concentrated within the rough microstructural parts, and this effect was more pronounced as the pressure increased. On the one-sided structure film, the stress was concentrated on one side of the microstructure and distributed over the entire surface of the other side. However, for films without microstructures, the stress was distributed over the entire top and bottom surface. In addition, Figure 2b shows the deformation of different microstructure films under different pressures. Similar to the results shown in Figure 2a, the three levels of microstructural deformation, in terms of size, were as follows: double-sided, one-sided, and non-structured. The differences in deformation were the result of stress concentrations in the microstructure; the smaller the area of stress, the greater the stress on the object for the same force, and therefore the greater the degree of deformation. The maximum compressive deformation of the double-sided microstructure film under 100 kPa force reached 30%, whereas the deformation of the film without a microstructure was less than 1%. Theoretically, the introduction of double-sided microstructure extensively improved the compression properties of the film and had the best effect on improving the sensor sensitivity.

### 3.2. Morphological and Structural Characterizations

#### 3.2.1. SEM Morphology of Sandpaper Microstructure

**Figure 3:**
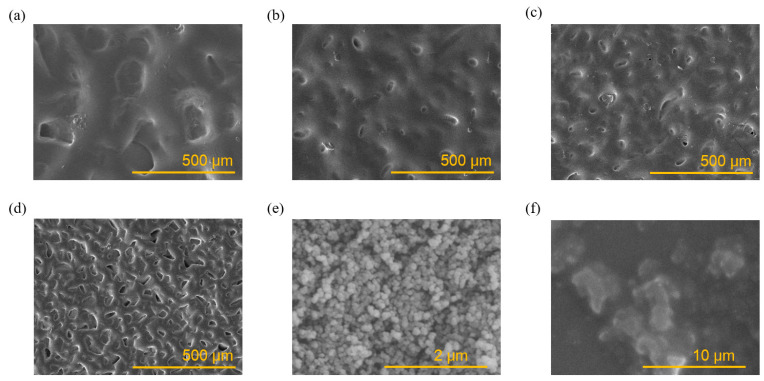
SEM morphologies of the surface of the PDMS film transferred with different grits of sandpaper: (**a**) 200#, (**b**) 400#, (**c**) 600#, (**d**) 800#, (**e**) CB (carbon black), and (**f**) the CB/PDMS composite film.

The main methods used to enhance the sensitivity of sensors are high-performance materials as well as a microstructure design. The sandpaper transfer method is a simple and effective microstructure construction method and is able to construct a rough surface on the surface of the flexible material, thus reducing the contact area and improving material compression properties. In this study, a simple and ingenious approach was taken to construct sandpaper microstructures on both sides of the PDMS film to enhance the sensor performance. Different particle sizes of sandpaper will result in different microstructure sizes and mechanical effects, and they have an indeterminate effect on the transfer and release process. Figure 3 shows the SEM morphology of the surface of PDMS transferred with different grits of sandpaper, including 200#, 400#, 600#, and 800#. It can also be seen that the microstructure particle size of sandpaper was larger at low mesh sizes, and the size of the microstructures formed by 200 mesh sandpaper was around 100 µm. As the mesh size of the sandpaper increased, the microstructure particle size of the sandpaper became smaller, the size of the microstructure fell to 25 µm, and the distribution within the unit area increased. The SEM morphologies of the carbon black and the CB/PDMS composite film are shown in Figure 3e,f.

#### 3.2.2. Mechanical Properties of Microstructures with Different Mesh Sizes

**Figure 4:**
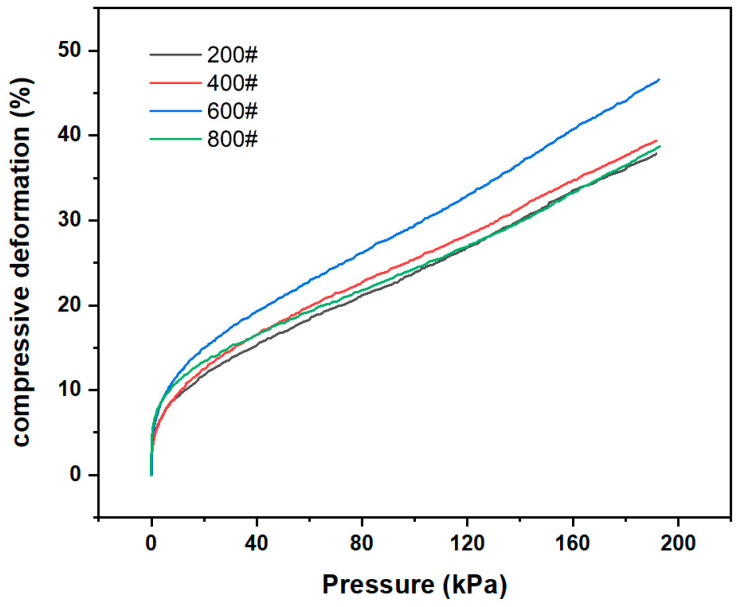
Compression properties of microstructure PDMS films with different mesh sizes.

Figure 4 illustrates the different compression properties of PDMS film with different mesh sizes under different pressures. The film transferred from the 600# sandpaper exhibited optimal compression properties. It can be seen the compression property of the film was enhanced with an increase in mesh size. But when the mesh number exceeded 600, the compression property of the film decreased instead. One possible reason for this is that at lower mesh sizes, the microstructures were larger and less distributed per unit area, thus limiting their compressibility. As the number of mesh increased, the size of each microstructure decreased as the total number of microstructures increased, so the force borne by each microstructure became smaller, and less force was required to make the film undergo compressive deformation. As the number of mesh increased further, the size of each microstructure decreased, and a single microstructure was no longer sufficient to undergo deformation, the compression performance of the overall film was diminished. In conclusion, the sensor constructed in this study employed a 600-mesh grit sandpaper to transfer the PDMS layer, thereby ensuring optimal compression properties and the highest sensitivity for the pressure sensor.

### 3.3. Performance Characterization of Pressure Sensor

#### 3.3.1. Sensor Structure and Sensing Mechanism

**Figure 5:**
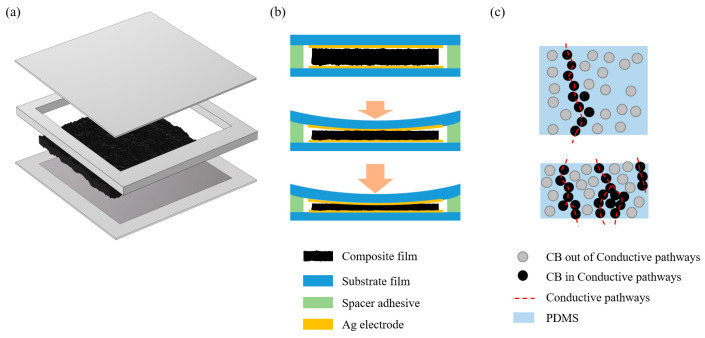
Sensor structure and sensing mechanism: (**a**) sensor structure diagram, (**b**) contact resistance effect, and (**c**) conductive pathways.

Figure 5 shows the structure and sensing mechanism of the pressure sensor. Figure 5a shows that the composite PDMS/CB film was sandwiched between the top and bottom electrodes, and there was also a layer of spacers around the film, connecting the top and bottom electrodes. This spacer layer has two functions. Firstly, it has adhesive upper and lower surfaces to encapsulate the device; secondly, the spacer has a similar height to the PDMS/CB composite film to maintain the initial state of the PDMS/CB film.

The sensing mechanism of the pressure sensor could be divided into two steps: a lower-pressure range and a higher-pressure range. The lower-pressure range was dominated by the contact resistance effect, while the higher-pressure range was dominated by the conductive pathways effect. It can be seen from the top graph of Figure 5b that in the initial state, the upper and lower electrodes did not exhibit significant contact with the intermediate conductive layer, so the overall resistance of the device was considerable. Once the pressure was applied, the upper and lower electrodes were in contact with the intermediate conductive layer, and a conductive path consisting of the bottom electrode, intermediate layer, and top electrode was generated. Meanwhile, due to the microstructure of the PDMS/CB layer, the ohmic contact between the electrodes and the rough microstructure of the surface became more complete as the pressure increased, which in turn resulted in a further reduction in the contact resistance between the electrodes and the interlayer. This effect significantly enhanced the sensitivity of the sensor.

As the applied pressure increased and approached the high-pressure range, the primary rationale for the sensor’s response could be attributed to the conductive pathways principle. When subjected to pressure, the CB particles within the PDMS were compressed, resulting in a reduction in the distance between them. This created longitudinal conductive loops between the CB particles in close proximity, based on the tunneling effect. As the pressure was increased further, the distance between the CB particles was additionally decreased, thereby generating additional conductive pathways and resulting in a further reduction in the loop resistance.

#### 3.3.2. Sensor Performance

**Figure 6:**
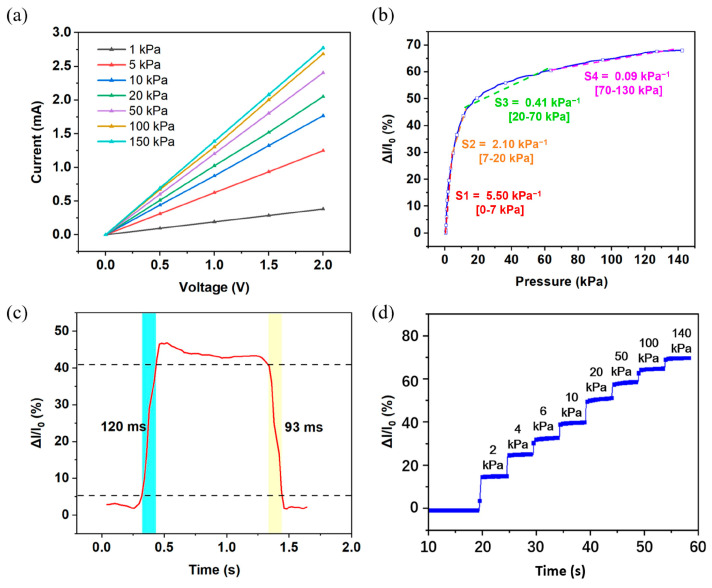
Electromechanical properties of the pressure sensor: (**a**) I–V curves under different pressure amounts; (**b**) sensitivity and sensing range of pressure sensor; (**c**) response and recovery time; and (**d**) current change under different pressure amounts.

The sensing properties of the pressure sensor were characterized by means of a testing system comprising a force gauge, a source meter, and an oscillometer. During measurements, the sensor was fixed at the platform of the force gauge, and pressure was applied through a 20 mm diameter probe. The real-time change in electronic signals was recorded using a source meter (Keithley 2400). Figure 6a shows the I-V curves of the pressure sensor under different pressures from 1 kPa to 150 kPa. As the pressure increased, the slope of the image also increased, indicating that the sensor resistance underwent a significant change in response to pressure. Furthermore, the sensor resistance was observed to decrease monotonically with pressure. In addition, the images also show that the sensor had good linearity.

The current change in the pressure sensor under different pressures was calculated and is shown in Figure 6b. The sensor sensitivity can be calculated by the following formula: S = (ΔI/I_0_)/ΔP, where I_0_ is the initial current, ΔI is the change in current, and P is the value of applied pressure. It can be seen that the sensitivity of the sensor was divided into three main typical intervals. In the range of 0–7 kPa, the sensor reached a high sensitivity of 5.5 kPa^−1^, which is mainly attributed to the double-sided microstructure of the PDMS/CB. The pressure sensor’s sensitivity was estimated to be 2.10 kPa^−1^ within the range of 7 to 20 kPa. At the pressure of 20–70 kPa, the sensitivity of the pressure sensor was estimated to be 0.41 kPa^−1^. As the pressure increased further over 70 kPa, the sensitivity of the sensor dropped to 0.09 kPa^−1^, but did not exhibit saturation. This is due to the fact that in large pressure intervals, additional conductive pathways were created within the PDMS/CB under pressure, thereby maintaining the observed trend of decreasing resistance. In conclusion, the sensor exhibits high sensitivity across a broad range of 140 kPa, thereby demonstrating that the microstructure design of the sensor is responsible for its excellent performance.

The response and release time of the pressure sensor was also characterized under a pressure of 10 kPa. The response and recovery times, shown in Figure 6c, were 120 ms and 93 ms, respectively. The current change in the pressure sensor during the loading/unloading experiment revealed a real-time response ability, which is crucial for the sensor to be utilized in real-time application scenarios.

Figure 6d shows the current change in the pressure sensor under different pressures, with the square wave pressure ranging from 0 to 140 kPa. It can be observed that when a constant pressure was applied to the sensor, the current change remained stable throughout the application of the pressure. Furthermore, it is evident that there was a clear difference in the current change for various static forces. The above result indicates that the pressure sensor has properties of high resolution and high stability.

**Figure 7:**
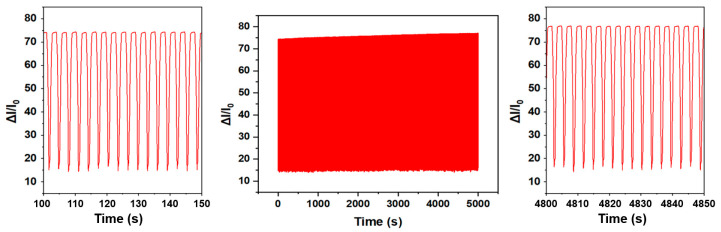
Stability of the pressure sensor.

The cyclic stability of the transducer under a load of 100 kPa was characterized under a gauge pressure tester, as shown in Figure 7. The current change rate of the pressure sensor was maintained stable after more than 2000 cyclic load tests. Compared with the beginning and ending cycles of the tests, the trend of the signal remained essentially unchanged, thus indicating that the sensor maintains reliable performance over an extended period of time.

The performance comparison between our pressure sensor and previously reported thin-film pressure sensors is listed in Table 1. From the results, one can conclude that our pressure sensor offers an optimal balance between sensitivity and range. For this ultra-thin type of pressure sensor, the construction of microstructures represents an effective method for enhancing the sensor’s performance. Nevertheless, the construction of microstructures on ultra-thin devices presents a significant challenge. Printed thin-film sensors exhibit low sensitivity and range, fabric and fiber sensors demonstrate low range, solution methods are challenging to adapt for microstructure fabrication, and transfer methods are straightforward but require the selection of an appropriate template.

The reason for the excellent performance of our sensors is the double-sided microstructure PDMS/CB composite film. In a low-pressure range, the contact resistance effect between the interlayer and the electrode layer endows the sensor with high sensitivity. In the high-pressure range, the longitudinal conductive pathways effect enables the sensor to further reduce its resistance under compression, thereby allowing our sensors to combine high sensitivity with a wide measuring range.

### 3.4. Application of the Pressure Sensors

Sitting posture is of great importance in the context of daily life. Adopting an optimal sitting posture not only helps prevent adverse health consequences but also enhances the comfort and effectiveness of work and study activities. Thin-film pressure sensors are instrumental in the detection of sitting posture, enabling real-time monitoring of the user’s sitting status and the provision of prompt feedback on poor sitting posture. This enables the prevention of health issues associated with poor sitting posture. Detecting sitting posture requires sensors that are characterized by a thin and lightweight form factor, coupled with high sensitivity, a broad sensing range, and a rapid response time. This enables the accurate capture of changes in sitting pressure in real time, facilitating adaptation to diverse cushion and seat shapes. Moreover, the sensors must exhibit stability to ensure long-term reliability.

**Figure 8:**
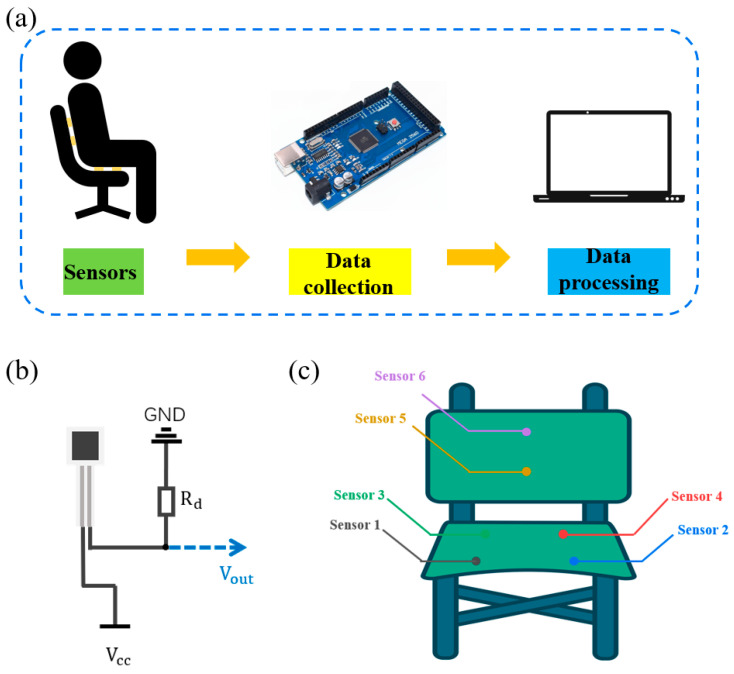
Sitting posture detection system diagram: (**a**) detection system diagram, (**b**) signal conversion circuit, and (**c**) the sensor’s placement on the seat.

Therefore, in this study, a sitting posture detection system was developed based on film pressure sensors to monitor the sitting posture in real time. The detection system framework diagram is shown in Figure 8a. The system consisted of a chair attached with six pressure sensors to detect external pressure, an MCU to acquire and transmit pressure signals, and a computer to receive signals. All six pressure sensors were connected to MCU through a voltage conversion circuit shown in Figure 8b. Figure 8c illustrates the distribution of sensors on the chair. Of these, sensor 1 to sensor 4 were affixed to the chair’s sear area to detect the support of the chair on the hips and thighs. Sensor 5 and sensor 6 were affixed to the chair’s back to detect the support of the chair’s back on the lumbar and dorsal regions. The working principle of the detection system is as follows. The sensors are initially calibrated to zero when the chair is in a state of no pressure. As the user assumes different postures, the contact between the sensors and the user’s body will result in different signal changes. By analyzing the pressure data from all the sensors, it is possible to determine the degree of support provided by the chair to different parts of the body in various sitting postures. This allows the user to distinguish between the good and bad sitting positions.

**Figure 9:**
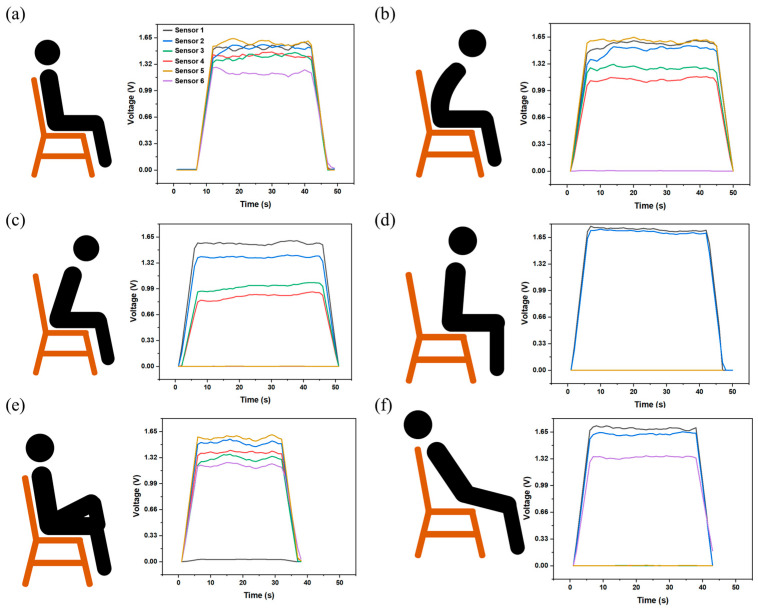
Different sitting postures and corresponding signals: (**a**) normal; (**b**) hunchback; (**c**) lean forward; (**d**) sit halfway; (**e**) cross legs; and (**f**) lie down.

Figure 9 shows all the sitting positions and the corresponding sensor signals. Figure 9a shows that, in good sitting postures, all body parts received equal and appropriate support from the chair. As shown in Figure 9b, when an individual sat in a hunchback posture, the signal from sensor 6 no longer existed, indicating that the back region was unsupported. Moreover, the signal of sensor 5 was significantly larger than other sensors, which means the lumbar region suffers from compression. Figure 9c represents an individual in a forward-leaning posture. The absence of a signal from sensors 5 and 6 suggests that the individual’s back and lumbar region were not adequately supported. Conversely, the signal of sensors 1 and 2 was notably higher than that of sensors 3 and 4, indicating that the forward-leaning posture compressed the thighs and may impede blood flow to the legs. Figure 9d shows a half-sitting posture, which, according to the sensor data, is a posture in which the lower back and thighs are in a suspended position, and where too much pressure is packed on the buttocks. Figure 9e illustrates a crossed-legged posture, which results in the thighs being positioned in a position of overhang and an uneven distribution of support from the chair compared with normal sitting posture. The last sitting posture, illustrated in Figure 9f, is a reclining posture. As indicated by the sensor signals, this position results in the hips and lower back being in a state of suspension, while simultaneously causing compression of the back and thighs.

In conclusion, the pressure sensors have a wide sensing range and they are thin, lightweight, highly sensitive, durable, and can be used in a range of applications such as human sitting posture detection.

## 4. Conclusions

In this study, a novel sandpaper transfer method was used to fabricate a double-sided microstructure conductive composite film, and then a sandwich structure thin-film pressure sensor was prepared by sandwiching the thin-film package with upper and lower electrodes. Owing to the superb mechanical properties of the composite film, the pressure sensor exhibited high sensitivity (5.5 kPa^−1^), a wide sensing range (0–140 kPa), a low response, and a short recovery time (120 ms/93 ms); it was also found to maintain good durability in more than 2000 cycle tests. Moreover, a sitting detection system based on the pressure sensors was developed to analyze the sitting forces and distinguish between healthy and unhealthy sitting postures. These results indicate that the sensor has the potential to be used in a wide range of application scenarios.

## Figures and Tables

**Table 1:** Performance comparison between our sensor and previously reported thin-film pressure sensors.

Material	Structure	Sensitivity (kPa^−1^)	Response Range (kPa)	Ref.
MXene/graphene	Film	2.13	10	[37]
PDMS/PEDOT:PSS	Sandpaper	2.32	100	[38]
PDMS/SnSe_2_/MWCNTs	Lotus leaf	165	80	[39]
LIG	Film	1.7	6	[40]
LIG	Microspheres	50	0.5	[41]
Ag particle	Fiber	4.55	10	[42]
PEDOT:PSS	Textile	6445	60	[43]
MXene/SWCNT	Spheres	2.6	7	[44]
PDMS/CB	Sandpaper	5.5	140	This work

## Data Availability

The data that support the findings of this study are available from the corresponding author upon reasonable request.
